# Airway Relaxation Effects of Water-Soluble Sclerotial Extract From *Lignosus rhinocerotis*

**DOI:** 10.3389/fphar.2018.00461

**Published:** 2018-05-07

**Authors:** Mei Kee Lee, Xiaojie Li, Alvin Chee Sum Yap, Peter Chi Keung Cheung, Chon Seng Tan, Szu Ting Ng, Richard Roberts, Kang Nee Ting, Shin Yee Fung

**Affiliations:** ^1^Department of Biomedical Science, University of Nottingham Malaysia Campus, Semenyih, Malaysia; ^2^School of Life Sciences, The Chinese University of Hong Kong, Shatin, China; ^3^Medicinal Mushroom Research Group, Department of Molecular Medicine, Faculty of Medicine, University of Malaya, Kuala Lumpur, Malaysia; ^4^LiGNO Biotech Sdn Bhd, Balakong Jaya, Malaysia; ^5^School of Life Sciences, University of Nottingham Medical School, Queen’s Medical Centre, Nottingham, United Kingdom; ^6^Center for Natural Products Research and Drug Discovery, University of Malaya, Kuala Lumpur, Malaysia

**Keywords:** tiger milk mushroom, *Lignosus rhinocerotis*, bronchorelaxation, airway diseases, aqueous extract, fractions, medicinal mushroom

## Abstract

*Lignosus rhinocerotis* has a long history of use by the indigenous community within East Asia to treat a range of health conditions including asthma and chronic cough. To date, there is limited scientific evidence to support its therapeutic effects in relieving these airways conditions. In this study, we examined the effects of the different molecular weight fractions [high-molecular-weight (HMW), medium-molecular-weight (MMW), and low-molecular-weight (LMW)] obtained from the cold water sclerotial extract (CWE) of *L. rhinocerotis* on airways patency using airway segments isolated from Sprague Dawley rat in an organ bath set-up. It is demonstrated that the HMW and MMW fractions exhibited higher efficacy in relaxing the pre-contracted airways when compared to the CWE and LMW fraction. In addition, the HMW fraction markedly supressed carbachol-, 5-hydroxytrptamine-, and calcium-induced airway contractions. CWE demonstrated a lower efficacy than the HMW fraction but it also significantly attenuated carbachol- and calcium-induced airway contractions. Results showed that the bronchorelaxation effect of CWE and fractions is mediated via blockade of extracellular Ca^2+^ influx. The composition analysis revealed the following parts of carbohydrate and proteins, respectively: HMW fraction: 71 and 4%; MMW fraction: 35 and 1%; and LMW fraction: 22 and 0.3%. Our results strongly suggest that the polysaccharide–protein complex or proteins found in the HMW and MMW fractions is likely to contribute to the bronchorelaxation effect of CWE.

## Introduction

*Lignosus rhinocerotis* (Cooke) Ryvarden, belonging to the *Polyporaceae* family, is commonly used as folk medicine among indigenous people in Southeast Asia to treat a variety of conditions, including cough, asthma and cancers (Abdullah et al., 2013). It is also known as *Polyporus rhinocerus* (Cooke), which is the taxonomical synonym of *L. rhinocerotis* (Huang, 1999). From ethnobotanical surveys, the methods of consumption reported are by boiling the mushroom sclerotium in water and drinking the decoction, or by eating the sclerotium raw (Azliza et al., 2012). The medicinal value of this mushroom is derived from its sclerotium.

Scientific evidence of the biological activity and bioactive components of *L. rhinocerotis* has been on the rise in recent years. The antiproliferative effect against several human leukemic cell lines (HL-60, K562 and THP-1) is suggested to be attributed to the hot water-soluble polysaccharide–protein complex extracted from the *P. rhinocerus* (Lai et al., 2008). Since the successful cultivation of *L. rhinocerotis* on agar, solid and spawn media in year 2009 (Tan, 2009), more chemical profiling and characterization of the bioactive components have been published. Evidence gathered from a number of different assays measuring the anti-inflammatory, anti-proliferative, and immunomodulatory activities of *L. rhinocerotis* consistently attributed its actions to the HMW components, in particular the polysaccharide–protein complex or proteins, fractionated from water-soluble sclerotial extract of *L. rhinocerotis* (Lee et al., 2012, 2014; Lau et al., 2013). Shotgun proteomics analysis of *L. rhinocerotis* has revealed that lectins and serine proteases are the most abundant proteins present in the sclerotium, together with other proteins including immunomodulatory protein 8 and cerato-platanin (Yap et al., 2015). Fungal immunomodulatory protein, FIP-fve, isolated from *Flammulina velutipes* was shown to attenuate the infiltrating inflammatory cells in the lungs of allergic asthma mouse model and reduced the pulmonary resist-ance measured by whole-body plethysmography (Lee et al., 2013).

Asthma and chronic obstructive pulmonary disease (COPD) are respiratory diseases characterised by airway obstruction, with varying degrees of inflammation (Cukic et al., 2012). One distinguishable physiological characteristic between the diseases is the reversibility of airflow limitation, with asthma being reversible and COPD being incompletely reversible, either spontaneously or with treatment. Bronchodilators, including β_2_-adrenoceptor agonists and muscarinic receptor antagonist, are the mainstay of treatment for asthma and COPD respectively (Proskocil and Fryer, 2005). One issue concerning the use of β_2_-adrenoceptor agonists is its diminishing effectiveness due to β_2_-adrenoceptor desensitisation after prolonged monotherapy (Cooper et al., 2011). Inhaled corticosteroids are often co-administered to treat underlying airway inflammation and to prevent tolerance to β_2_-adrenoceptor agonists (Barnes and Adcock, 2003). Moreover, development of an effective antimuscarinic bronchodilator has been challenging to balance between clinical effectiveness and side effects of the drug (Moulton and Fryer, 2011). The currently available bronchodilators are not ideal, hence, the need to search for alternative therapies with different mechanisms of action.

The pathophysiology of asthma and COPD has been linked with excessive release of acetylcholine, causing overactivation of muscarinic receptors and subsequently airway smooth muscle contraction (Erle and Sheppard, 2014). Serotonin or 5-hydroxytryptamine (5-HT) is another neurotransmitter which has complex actions on pulmonary function. Activation of 5-HT receptors on airway smooth muscle has been reported to cause smooth muscle contraction or relaxation. Another explanation for the complex action of 5-HT is that its presence could indirectly modulate the acetylcholine level leading to muscle contraction (Cazzolau et al., 1995). In rat, 5-HT activates 5-HT_2A_ receptors on airway smooth muscle and causes airway contraction. In human, 5-HT plasma levels are elevated in asthmatic patients and *in vitro* evidences showed that it might have a role in asthma (Dupont et al., 1999). Activation of G_αq_-protein-coupled M_3_ and 5-HT_2A_ receptors on airway smooth muscle, results in 1, 4, 5-trisphosphate (IP_3_) and diacylglycerol (DAG) generation and mobilisation of extracellular and intracellular stored Ca^2+^. Elevation of cytoplasmic Ca^2+^ level subsequently leads to airway smooth muscle contraction (Cazzolau et al., 1995; Erle and Sheppard, 2014).

To date, no other group has reported the direct effect of airway relaxation mediated by the bioactive components of *L. rhinocerotis*. The objectives of this study were to examine the airway relaxation effect of the different molecular weight fractions obtained from the CWE of *L. rhinocerotis* and to investigate the mechanism of the bronchodilator action.

## Materials and Methods

### Preparation, Extraction and Fractionation of the Sclerotial Cold Water Extract

Ground mushroom sclerotial powder (0.2 mm sieve) from *L. rhinocerotis* (TM02), identified by the internal transcribed spacer (ITS) regions of their ribosomal RNA (Tan et al., 2010), was supplied by LiGNO Biotech Sdn Bhd (Selangor, Malaysia). The mushroom specimen voucher is deposited in the Royal Botanic Garden Kew, K(M)177812. The preparation method for the CWE was adopted from our previous paper (Yap et al., 2013). In brief, the sclerotial powder was extracted by stirring in cold water at 4°C for 24 h at a ratio of 1:20 (w/v). CWE obtained by filtration through Whatman No. 1 filter paper was then freeze-dried. CWE was re-dissolved in purified water (Milli-Q quality) to make a stock solution of 100 mg/ml before being fractionated by gel permeation chromatography using a Sephadex G50 column (212 ml). Aqueous elution was carried out at room temperature by gravity and 100 fractions of 2.5 ml volume each were collected. Aliquots from the tubes were sampled for testing the protein and carbohydrate content by measurement of tis absorbance reading at 595 and 490 nm, respectively. Molecular weight (MW) of the pooled fractions was determined by comparing their ratio of elution volume (Ve) and void volume (Vo) to the Ve/Vo of selected protein standards including carbonic anhydrase (29 kDa), soybean trypsin inhibitor (20 kDa), ribonuclease A (14 kDa), cardiotoxin (7 kDa), cobalamin (1.35 kDa), and riboflavin (0.376 kDa).

### Determination of Total Carbohydrate and Protein Contents

Carbohydrate and protein contents in the different fractions were determined with phenol-sulfuric acid method as described by DuBois et al. (1956) and Bradford (1976) method, respectively. D-Glucose and bovine serum albumin (BSA) were used as standards, respectively.

### Determination of Monosaccharide Composition

The monosaccharide composition of the carbohydrate moiety in the CWE and its different MW fractions were determined by the alditol acetate derivatives of the monosaccharides by gas chromatography (7890B, Agilent Technology, United States) equipped with an Alltech DB-225 capillary column (15 m × 0.25 mm i.d., 0.25 μm films). The oven temperature was increased from 150 to 220°C at the rate of 2°C/min and kept at 220°C for 15 min. The carrier gas was helium. The monosaccharide standards consisted of D-fucose, L-rhamnose, D-ribose, L-arabinose, D-xylose, D-mannose, D-galactose, and D-glucose. D-Allose was used as internal standard. All the monosaccharide standards were purchased from Sigma-Aldrich, United States.

### Tissues Preparation

Ethical approval was obtained from the University of Nottingham’s Animal Welfare and Ethics Review Body (UNMC#2kn and UNMC12). All applicable international, national and/or institutional guidelines for the care and use of animals were followed. Experiments were conducted with male Sprague Dawley rats (235–440 g; 2–3 months old). Sprague Dawley rats were purchased from University Putra Malaysia or University Kebangsaan Malaysia and sacrificed on the day of experiment. Rat trachea and bronchus were removed, excised into 2 mm rings, and immediately immersed in standard Krebs-Ringer bicarbonate solution (pH 7.4). All the methods used in this study were adopted from previous study (Loong et al., 2015). Changes in muscle tension responses produced by tissue contraction and relaxation were detected by force transducer (MLTF050/ST, ADInstruments, United States). Data were recorded by a PowerLab data acquisition system (LabChart v7.3.4) and a computer (Hewlett-Packard, United States). Muscle tension was expressed in milliNewtons (mN). Tissues were left to equilibrate to bath conditions for 30 min after the application of 9.8 mN tension to achieve a steady baseline tone. Following equilibration, all tissues were exposed twice to 60 mM potassium chloride (KCl) to assess tissue viability and to provide reference contractions for subsequent data analysis. After the KCl was washed out and a stable basal tone was re-established, experiments were carried out as in Section “Organ Bath Experimental Protocol.”

### Organ Bath Experimental Protocol

#### Concentration-Response Curves to CWE and Fractions Against Pre-contracted Airway Segments

Tracheal and bronchial segments were pre-contracted with carbachol (1 μM), a non-selective acetylcholine receptor agonist, to obtain a stable submaximal contraction between 70 and 80% of the maximum carbachol contraction [determined from previous experiments (data not shown)]. Once a stable contraction was established, cumulative concentrations of CWE, HMW, MMW, or LMW fractions, was added into the preparation every 15 min. The degree of response (tissue relaxation) was measured as a fraction of the contraction achieved by 1 μM of carbachol. The tissue relaxation was expressed as a percentage of carbachol-induced tone (*n* = 4 to 6 animals). Vehicle control, either purified water or the appropriate concentration of DMSO, was carried out in all the following protocols.

#### Effect of CWE and Fractions Against Carbachol and 5-Hydroxytrptamine (5-HT) Concentration-Response Curve

Tracheal rings were pre-incubated with 2.5 mg/ml CWE, 2.5 mg/ml HMW fraction, 2.5 mg/ml LMW fraction, or 30 nM ipratropium (muscarinic receptor antagonist) for at least 30 min, prior to the construction of the carbachol or 5-HT concentration-response curve (CRC). Tissue contraction to carbachol or 5-HT was expressed as a percentage of 60 mM KCl-induced tone (*n* = 5 to 6 animals).

#### Effect of CWE and Fractions on Calcium-Induced Contraction

Tracheal segments were bathed in calcium-free Krebs solution and pre-incubated with the 2.5 mg/ml CWE, 2.5 mg/ml HMW fraction, 2.5 mg/ml LMW fraction, or 10 μM nifedipine for at least 30 min. KCl (60 mM) was added to depolarize the cell membrane and left to incubate for 10 min before calcium chloride (CaCl) was reintroduced cumulatively. Tissue contraction to CaCl was expressed as a percentage of the 60 mM KCl-induced tone (*n* = 5 to 7 animals).

### Statistical Analysis

Data were analysed and graphs were drawn using PRISM v7.0 (GraphPad software). All data were expressed as mean ± standard deviation (SD) of *n* number of animals. Maximum tissue contraction or relaxation response (E_max_) was derived from non-linear regression analysis of the obtained CRC or from the response generated at the highest concentration tested. Statistical analyses were performed using one-way ANOVA, two-way ANOVA with Bonferroni correction and Student’s unpaired *t*-test. Results were considered statistically significant if *p*-value was less than 0.05.

### Drugs

Carbamycholine chloride or carbachol (Nacalai Tesque, Japan), 5-hydroxytryptamine (Nacalai Tesque, Japan), and ipratropium bromide (Tocris, United Kingdom) were dissolved in purified water. Nifedipine (Nacalai Tesque, Japan) was dissolved in 100% DMSO (Sigma-Aldrich, United States) to make 100 mM of stock solution and further diluted in 50% DMSO to make 10 mM of nifedipine solution.

## Results

### Fractionation of Sclerotial Extract From Lignosus rhinocerotis

**FIGURE 1:**
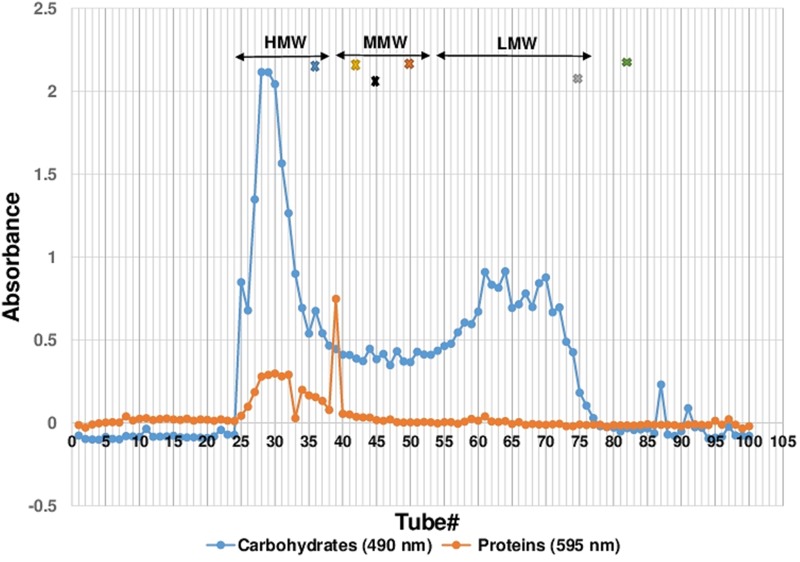
Sephadex^®^ G-50 Fractionation of CWE. Fractionation of 100 tubes were collected with each tubes containing 2.5 mL of fraction. Proteins and carbohydrates content were measured as absorbance reading at 595 and 490 nm, respectively. The fractionation of the CWE was separated into three molecular weight ranges, HMW, MMW and LMW. Protein standards used for determining the molecular mass of pooled fraction were carbonic anhydrase (29 kDa), soybean trypsin inhibitor (20 kDa), ribonuclease A (14 kDa), cardiotoxin (7 kDa), cobalamin (1.35 kDa), and riboflavin (0.376 kDa) as indicated as x in the colour of blue, black, gold, orange, grey and green, respectively. CWE, *Lignosus rhinocerotis* TM02 sclerotial CWE.

Cold water extraction of the sclerotial powder of *L. rhinocerotis* yielded 7.04 ± 0.69 g of freeze-dried solid. Further fractionation of the extract by Sephadex^®^ G-50 gel filtration chromatography yielded a broad protein peak and several carbohydrate peaks (**Figure 1**). The fraction tubes collected were pooled into three fractions based on their molecular weight range. They are the high-, medium-, and low-molecular-weight fractions designated by HMW, MMW, and LMW, respectively. The MW range of each fraction were: HMW (>29.9 kDa), MMW (7.1 to 27.4 kDa) and LMW (0.8 to 6.5 kDa).

### Chemical Composition of CWE and Its Fractions

The chemical composition in **Table 1** show that the majority of carbohydrate and protein present in CWE is found in the HMW fraction, indicating the presence of polysaccharides and polysaccharide–protein complexes. There was a larger amount of non-protein and non-carbohydrate substances in the LMW and MMW fractions than the HMW fraction. These are LMW molecules such as phenolic compounds found abundantly in CWE (Yap et al., 2013). The monosaccharide profile of CWE and its fractions indicated the presence of different MW carbohydrates having different sugar composition. Glucose was the most dominant sugar found in CWE and its fractions indicating the presence of water-soluble glucans which are commonly found in mushrooms. Other than glucans, there were small amounts of other heteroglycans containing arabinose, mannose and galactose in CWE and its fractions. It was noted that the amount of mannose and galactose in the fractions correlated with the amount of protein in the fractions, suggesting the presence of manno- and/or galacto-glycoprotein linkages in polysaccharide–protein complexes.

**Table 1:** Chemical composition and monosaccharide profile of CWE and its fractions.

Sample	Protein %	Total carbohydrate %	Monosaccharides (normalised %)				
			Arabinose	Xylose	Mannose	Galactose	Glucose
TM02 sclerotial powder	1.38	7.76	-	2.39	2.52	1.73	93.37
CWE	2	68	0.39	-	1.25	2.29	96.08
LMW	0.3	22	3.11	-	5.39	-	91.49
MMW	1	35	-	-	-	1.25	98.74
HMW	4	71	-	-	5.11	4.62	90.27

### Unequivocal Airway Relaxation Effects of CWE, HMW and MMW Fractions

**FIGURE 2:**
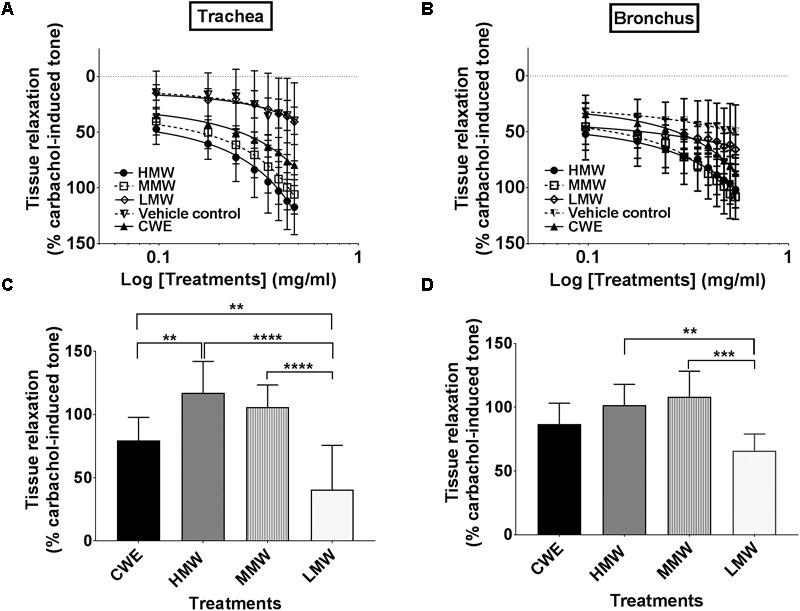
Effect of CWE and fractions cumulative CRC against carbachol-induced contractions of the rat isolated **(A)** trachea and **(B)** bronchus. Comparison of the bronchorelaxation effects of the fractions at the highest concentrations tested are depicted in **(C)** trachea at 3.0 mg/ml and **(D)** bronchus at 3.5 mg/ml. Tissue responses have been expressed as a percentage of the carbachol-induced contraction and are shown as means ± SD of 4 to 6 animals. CWE, cold water extract; HMW, high-molecular-weight fraction; MMW, medium-molecular-weight fraction; LMW, low-molecular-weight fraction (two way ANOVA: ^∗∗^*p* < 0.01; ^∗∗∗^*p* < 0.001; ^∗∗∗∗^*p* < 0.0001).

CWE relaxed both tracheal (79.45 ± 39.68%, *p* = 0.0018) and bronchial (86.90 ± 16.30, *p* = 0.0119) segments and both responses were statistically different from the corresponding vehicle control (39.68 ± 5.47%) (see **Figures 2A,B**). Based on the maximum tissue relaxation (E_max_) (see **Figures 2C,D**), the effectiveness of the test samples is similar in trachea (T) and bronchus (B): HMW fraction (T: 117.32 ± 24.62%; B: 101.67 ± 16.34%) and MMW fraction (T: 105.84 ± 17.58%; B: 108.30 ± 19.98%) produced the largest response followed by CWE. The responses produced by the LMW fraction in trachea and bronchus were no different (T: 40.64 ± 35.02%; B: 65.80 ± 13.21%) from vehicle control (T: 39.68 ± 12.23%; B: 49.68 ± 23.70%).

### HMW Fraction Markedly Prevents Carbachol- and 5-HT-Induced Airway Constriction

**FIGURE 3:**
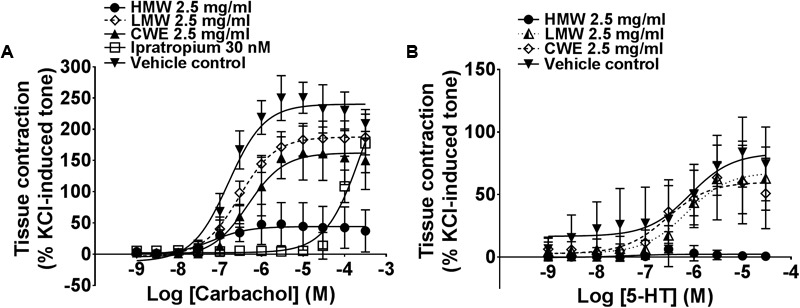
Effect of CWE and fractions pre-incubation against **(A)** carbachol and **(B)** 5-HT CRC in rat isolated tracheal rings. Concentration-response curves (CRCs) to **(A)** carbachol and **(B)** 5-HT in the absence or presence of CWE and fractions in rat isolated tracheal rings. Tissue responses have been expressed as a percentage of KCl-induced contraction and are shown as means ± SD of 5 to 6 animals.

Based on the above findings (see section “Unequivocal Airway Relaxation Effects of CWE, HMW and MMW Fractions”), subsequent experiments were carried out using 2.5 mg/ml of CWE, HMW and LMW fractions on tracheal rings. HMW fraction almost totally prevented carbachol- (E_max_: 44.52 ± 36.61%, *p* = 0.0002) and 5-HT-(E_max_: 2.48 ± 3.41%, *p* < 0.0001)-induced contraction compared to the vehicle control (E_max_ of carbachol: 241.20 ± 24.41%; E_max_ of 5-HT: 85.24 ± 30.95%) (**Figure 3**) in the airway segments. Although the effect of CWE (E_max_: 165.00 ± 45.37%, *p* = 0.0129) was not as marked as HMW fraction, it also significantly attenuated the E_max_ of carbachol and exhibited a similar response as ipratropium (E_max_: 177.30 ± 46.88%, *p* = 0.0447). On the contrary, LMW fraction did not affect any of the tissue contractions induced by carbachol (E_max_: 187.90 ± 26.88%, *p* = 0.1497) or 5-HT (E_max_: 69.91 ± 24.22%, *p* > 0.999). Results are summarised in **Table 2**.

**Table 2:** The maximum response (E_max_) of carbachol, 5-HT and CaCl derived from the respective concentration-response curve (CRC) in the presence of different treatment.

Treatments	Carbachol		5-HT		CaCl	
	*n*	E_max_ (%)	*n*	E_max_ (%)	*n*	E_max_ (%)
+ Vehicle control	6	241.20 ± 24.41	5	85.24 ± 30.95	7	91.44 ± 31.11
+ HMW (2.5 mg/ml)	5	44.52 ± 36.61^∗∗∗∗^	5	2.48 ± 3.41^∗∗∗^	5	37.75 ± 8.83^∗∗∗^
+ LMW (2.5 mg/ml)	5	187.90 ± 26.88	6	69.91 ± 24.22	5	100.90 ± 18.10
+ CWE (2.5 mg/ml)	6	165.00 ± 45.37^∗^	6	60.28 ± 31.64	5	54.48 ± 11.79^∗^
+ Ipratropium (30 nM)	6	177.30 ± 46.88^∗^	-	-	-	-
+ Nifedipine (10 μM)	-	-	-	-	5	21.83 ± 12.54^∗∗∗∗^

*Tissue responses have been expressed as a percentage of KCl-induced contraction and are shown as means ± SD. Unpaired *t*-test where comparison of the mean was made between vehicle control and the treatment group; ^∗^*p* < 0.05; ^∗∗∗^*p* < 0.001, ^∗∗∗∗^*p* < 0.0001. CWE, cold water extract; HMW, high-molecular-weight fraction; MMW, medium-molecular-weight fraction; LMW, low-molecular-weight fraction.*

### HMW Fraction Significantly Limits Calcium-Induced Contraction

**FIGURE 4:**
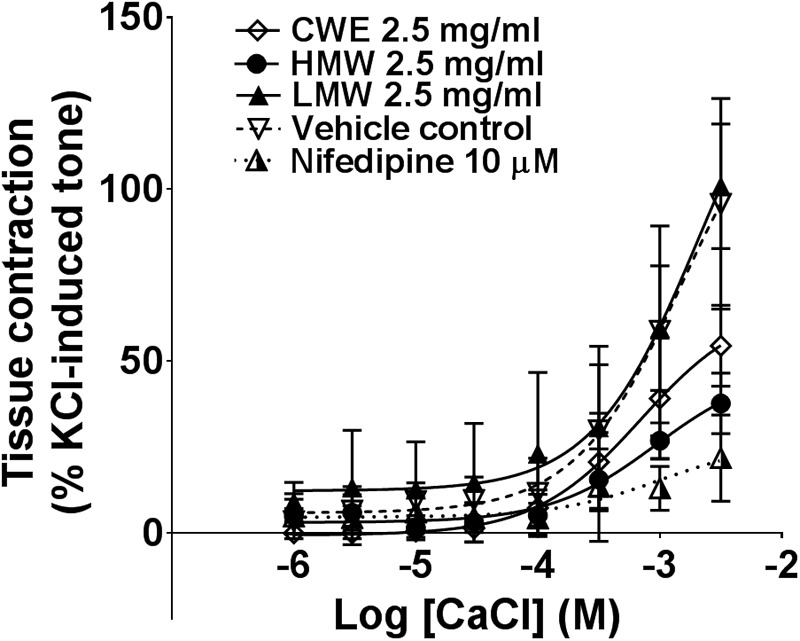
Effect of CWE and fractions against CaCl CRC in rat isolated tracheal rings. Concentration-response curves to calcium in the absence or presence of CWE and fractions in rat isolated tracheal rings. Tissue responses have been expressed as a percentage of KCl-induced contraction and are shown as means ± SD of 5 to 7 animals.

Smooth muscle contraction is associated with increased intracellular Ca^2+^ level. This is mediated via extracellular Ca^2+^ influx through membrane calcium channel or release of Ca^2+^ from intracellular sarcoplasmic reticulum stores. The HMW fraction (E_max_: 37.75 ± 8.83%, *p* = 0.0005) significantly attenuated the contraction induced by external calcium, similar to the effect of nifedipine (E_max_: 21.83 ± 12.54%, *p* = 0.0001) compared to vehicle control (E_max_: 91.44 ± 31.11%) (**Figure 4**). CWE (E_max_: 54.48 ± 11.79%, *p* = 0.0149) also significantly reduced the E_max_ of calcium chloride, although to a lesser extent than HMW fraction and nifedipine. The LMW fraction (E_max_: 100.90 ± 18.10%, *p* = 0.829) had the same effect as vehicle. It was unable to calculate the E_max_ from non-linear regression curve and therefore E_max_ was taken as the response at highest concentration tested. Results are summarised in **Table 2**.

## Discussion

*Lignosus rhinocerotis* has a long history of use by the indigenous community to treat asthma and chronic cough (Lau et al., 2015). This is the first study to elucidate the bioactive fractions accounting for the airway relaxation effect elicited by this mushroom. Our earlier study showed that CWE has fully relaxed both the isolated tracheal and bronchial segments pre-contracted with carbachol (Lee et al., 2018). Two most common bronchodilators used to reverse airways constriction act via stimulation of β_2_-adrenoceptors (e.g., salmeterol) or antagonism of muscarinic receptors (e.g., ipratropium). The relaxation effects of CWE are not mediated through β_2_-adrenoceptor stimulation, nitric oxide, or K^+^ channel (see Supplementary Figure 1 and Lee et al., 2018) or blockade of muscarinic receptors (see **Figure 3A**). Our evidence to date implies that CWE relaxes airways through a mechanism different from that used by the conventional bronchodilators. Interestingly, CWE significantly limits calcium-induced contraction in the tracheal rings. This implies that one of mechanisms of relaxation in the tracheal smooth muscle is via the blockade of external calcium entry similar to the effect exhibited by nifedipine, a L-type calcium channel blocker.

To identify the bronchodilator components, the CWE was fractionated using Sephadex G-50 gel filtration chromatography into HMW, MMW and LMW fractions. We have successfully demonstrated that both HMW and MMW fractions exhibited higher efficacy in relaxing the pre-contracted airways when compared to CWE and LMW fraction. This demonstrates that the bioactive components responsible for the airway relaxation effects are large molecular weight substances present mostly in the HMW and MMW fractions. Responses of the LMW fraction were similar to the vehicle control thus indicating no significant bioactive components are present in this fraction.

Activation of G_αq_-protein-coupled M_3_ and 5-HT_2A_ receptors leads to an overall increase in the intracellular Ca^2+^ free concentration which results in airway smooth muscle contractions. We showed that pre-incubation with the HMW fraction inhibits the contraction of the airways to both carbachol and 5-HT to a similar degree. The HMW fraction abolished the carbachol- and 5-HT-induced contractions in a non-competitive manner. This suggests that the HMW fraction non-selectively inhibits the downstream mechanism of activated G_αq_-coupled receptors. Our postulation is based on our earlier work which showed that carbachol-induced contraction in the trachea is largely dependent on extracellular Ca^2+^ entry (unpublished data) with little contribution from intracellular Ca^2+^ stores. Following these observations, we subsequently investigated the effect of the CWE and its fractions on external Ca^2+^ entry. In the absence of extracellular Ca^2+^, addition of KCl depolarizes the smooth muscle cell membrane and opens voltage-gated Ca^2+^ channels, resulting in an influx of Ca^2+^ upon calcium re-introduction. Nifedipine, a selective voltage-gated Ca^2+^ channel blocker, was chosen as a positive control in this study to confirm the involvement of these channels in the contraction elicited following re-introduction of calcium. The effectiveness of the HMW fraction in blocking the calcium-induced contraction was similar to nifedipine thus implying the HMW fraction may be blocking voltage-gated Ca^2+^ channels. This is supported by observations from an earlier calcium imaging study showing that CWE (likely HMW fraction as well) inhibits extracellular Ca^2+^ influx into rat dorsal root ganglion cells (Lee et al., 2018). Given this evidence, we conclude that one of the mechanisms by which the CWE induces airway relaxation is linked to regulation of calcium signalling in response to G_αq_-protein-coupled receptor activation. We are not excluding the possibility that the CWE or the HMW fraction might also influence other regulators that modulate smooth muscle contractility such as IP_3_, DAG, protein kinase C, and Rho kinase. The precise mechanisms of the CWE and its active fractions remain to be elucidated.

Our observations lead us to postulate that treatment with *L. rhinocerotis* may confer protection against acute respiratory tract exacerbations and that it has the potential to preserve airways patency. Our results also showed that the efficacy of 2.5 mg/ml CWE and HMW fraction is comparable to or even better than that of 30 nM ipratropium in inhibition of carbachol-induced airway contraction.

The biological activity of other types of medicinal mushroom, in particular the immunomodulatory activity, has been associated with the HMW polysaccharide or polysaccharide–protein complex found in the mushroom extract (Tzianabos, 2000; El Enshasy and Hatti-Kaul, 2013). Oral application of a polysaccharide fraction with a highly branched arabinogalactan protein derived from a medicinal plant, *Terminalia chebula*, displayed antitussive properties in TRIK strain guinea pigs (Nosalova et al., 2013).

In this study, the largest amount of carbohydrate and protein was found in the HMW fraction (71 and 4%, respectively), followed by the MMW fraction (35 and 1%, respectively) and the LMW fraction (22 and 0.3%, respectively). As the LMW fraction containing an insignificant amount of protein did not display any bronchodilator properties at the concentration tested our results strongly suggest that the polysaccharide–protein complex or proteins found in the HMW and MMW fractions are likely to contribute to the bronchorelaxation effects of the CWE. This is in agreement with another published study using the same TM02 cultivar where the HMW fraction exhibited the greatest anti-inflammatory activity and anti-proliferative properties against human lung and breast cancer cells (Lee et al., 2014).

Our monosaccharide profile analysis revealed that glucose is the most abundant sugar in the CWE and all its fractions. The amount of galactose and mannose corresponds with the bronchorelaxation activities; both sugars are found in the highest amount in the bioactive HMW fraction, followed by CWE. However, mannose does not seem to contribute to the bronchodilator effect as the MMW fraction is devoid of mannose but able to produce pronounced bronchorelaxation similar to the HMW fraction. Furthermore, mannose, but not galactose, is found in the LMW fraction. We postulate that the glucose and galactose consistently found in the HMW fraction, the MMW fraction, and the CWE appeared to be the important residues in the glycosidic linkages of the polysaccharide–protein complex contributing to the bronchorelaxation activities. Recently, a novel polysaccharide–protein complex was isolated from the hot-water-soluble polysaccharide fraction of *P. rhinocerus* and chemically characterised, indicating that glucose and mannose are the predominant carbohydrate moieties in the polysaccharide–protein complex with antitumor activity (Liu et al., 2016). The slight variations in the sugar composition between our study and the previous report might be attributed to the differences in extraction method, cultivar and geographical origins. In addition, in contrary with the previous findings by Liu et al. (2016), our preliminary experiments with the hot water extract of *L. rhinocerotis* have showed no bronchorelaxation effect (unpublished data), suggesting that the manno-glycoprotein linkages in the polysaccharide–protein complex are not likely to contribute to the bronchorelaxation effect.

## Conclusion

This is the first study to report the direct relaxation effects of active fractions derived from water-soluble sclerotial extract from *L. rhinocerotis* on airway segments from rats. This study confirms that the bioactive components are the HMW polysaccharide–protein complexes or proteins found in HMW and MMW fractions. The important sugar moieties are glucose and galactose. One of the mechanisms of relaxation is likely to be due to blockade of extracellular Ca^2+^ influx through the membrane calcium channels downstream of G_αq_-protein-coupled receptors. Further investigations are required to identify and elucidate the structure of the large molecular weight bioactive components from the sclerotium of *L. rhinocerotis* for their potential therapeutic application as bronchodilators.

## Author Contributions

SYF, KNT, RR, and PCKC conceived and designed the experiments. MKL, XJL, and ACSY performed the experiments and analyzed the data. STN, CST, KNT, PCKC, and SYF contributed reagents/materials/analysis tools. MKL, SYF, KNT, and PCKC wrote the paper. All authors agreed to be accountable for all aspects of the work in ensuring that questions related to the accuracy or integrity of any part of the work are appropriately investigated and resolved.

## Conflict of Interest Statement

LiGNO Biotech Sdn Bhd supplied the sclerotial powder of *Lignosus rhinocerotis*. The authors declare that the research was conducted in the absence of any commercial or financial relationships that could be construed as a potential conflict of interest.

## Abbreviations

CWE: cold water extract
HMW: high-molecular-weight
LMW: low-molecular-weight
MMW: medium-molecular-weight
