# Supplementary material for: Airway Relaxation Effects of Water-Soluble Sclerotial Extract From Lignosus rhinocerotis
Source: Front Pharmacol. 2018 May 7;9:461. doi: 10.3389/fphar.2018.00461 (PMC5949369; doi:10.3389/fphar.2018.00461)
Supplement: Supplementary file 1 [file Data_Sheet_1.docx]

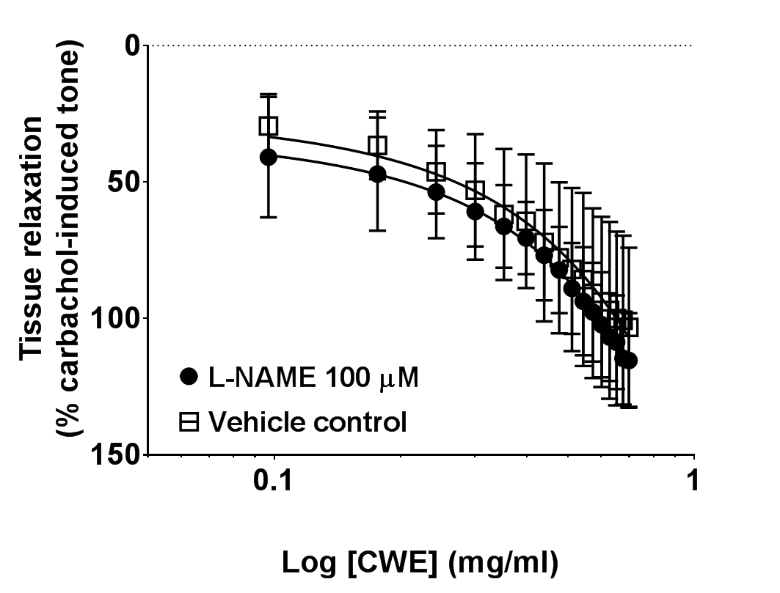


**Supplementary figure 1. Role of nitric oxide in CWE-induced airway relaxation in rat isolated trachea.** Tissues were pre-incubated with NG-nitro-L-arginine methyl ester (L-NAME), a nitric oxide synthase inhibitor, or vehicle control 30 minutes before contraction with 1 µM carbachol, followed by CWE cumulative CRC. Tissue responses have been expressed as a percentage of carbachol-induced contraction and are shown as means ± SD of 4 to 5 animals.
